# Recent Advances in the Metabolic Engineering of Yeasts for Ginsenoside Biosynthesis

**DOI:** 10.3389/fbioe.2020.00139

**Published:** 2020-02-25

**Authors:** Luan Luong Chu, Jake Adolf V. Montecillo, Hanhong Bae

**Affiliations:** Department of Biotechnology, Yeungnam University, Gyeongsan-si, South Korea

**Keywords:** ginsenosides, metabolic engineering, system approaches, fermentation, yeast

## Abstract

Ginsenosides are a group of glycosylated triterpenes isolated from *Panax* species. Ginsenosides are promising candidates for the prevention and treatment of cancer as well as food additives. However, owing to a lack of efficient approaches for ginsenoside production from plants and chemical synthesis, ginsenosides may not yet have reached their full potential as medicinal resources. In recent years, an alternative approach for ginsenoside production has been developed using the model yeast *Saccharomyces cerevisiae* and non-conventional yeasts such as *Yarrowia lipolytica* and *Pichia pastoris*. In this review, various metabolic engineering strategies, including heterologous gene expression, balancing, and increasing metabolic flux, and enzyme engineering, have been described as recent advanced engineering techniques for improving ginsenoside production. Furthermore, the usefulness of a systems approach and fermentation strategy has been presented. Finally, the present challenges and future research direction for industrial cell factories have been discussed.

## Introduction

Ginsenosides represent a group of glycosylated triterpenes found from the *Panax* species. To date, ~180 ginsenosides have been identified from around 17 species of the *Panax* genus (Liang and Zhao, 2008; Kim et al., 2015; Yang et al., 2018). *Panax* belongs to the Araliaceae family, which contains *Panax ginseng, Panax quinquefolius*, and *Panax notoginseng*, which have been used in traditional herbal medicine in Asia for more than 4,000 years (Duan et al., 2017; Mancuso and Santangelo, 2017). Notably, ginsenosides are also detected in *Pfaffia glomerata*, which is Brazilian ginseng belonging to the Amaranthaceae family (Costa et al., 2018). As major bioactive components of ginseng plants, ginsenosides have various beneficial effects on health, including antidiabetic, antioxidant, antimicrobial, antiplatelet, antitumor, anti-proliferative, and anticancer activities (Nag et al., 2012; Le et al., 2015; Park et al., 2017; Irfan et al., 2018; Kim et al., 2019; Zhou et al., 2019). In addition, ginsenosides play a significant role in not only enhancing central nervous system activities but also improving the protection of blood vessels from cardiovascular disease and modulating immune functions (Leung and Wong, 2010; Rajabian et al., 2019). Moreover, ginsenosides and their derivatives have been used in the food and cosmetic industries (Shi et al., 2010; Kim E. et al., 2018).

Although it has been reported that the world ginseng production has reached more than 80,080 tons, the mainly ginsenosides and its derivatives have extracted from *Panax* plants (Baeg and So, 2013; Rahimi et al., 2019). Regarding ginsenosides production, ginseng agriculture produces are limited yield, and therefore, ginsenosides is slow and difficult obtained through this method. This is because ginseng grows slowly and requires approximate 6 years from seedling to the harvesting of the roots to produce marketable ginseng. Additionally, many biological and environmental factors affect ginseng growth, such as cultivar type, diseases, herbivorous animals, soil type, and climatic factors. The highest ginsenoside accumulation is found in the root and root hair, and root growth depends on the season (Park et al., 2012; Kim et al., 2015). Noticeably, bioengineering strategies, including cell and tissue cultures, treatment of biotic and abiotic elicitors, and transgenic plants, have been successfully obtained to improve ginsenoside production. However, these methods still have their challenges, such as the biochemical instability of cell and tissue cultures and the low productivity of ginsenoside extraction (Roberts, 2007; Murthy et al., 2014; Kim et al., 2015). Furthermore, the chemical structures of ginsenosides are complex, leading to difficulties in chemical synthesis. These reasons made the price of ginsenosides considerably high. In 2018, more than billion USD worth of ginsenosides was consumed worldwide due to their numerous useful applications. In addition, it is predicted that the market for ginsenosides will be at around trillion USD by 2050 (Baeg and So, 2013; Rahimi et al., 2019).

The microbial biosynthesis of ginsenosides from renewable resources is a promising alternative strategy to address the constantly increasing demand for ginsenosides in recent years. Microbes possess many advantages compared to plant cells, such as requiring less land for growth, fast-growing with high cell density cultivation, controllable and well-characterized genetics, and well-developed genetic manipulation technology (Lee et al., 2012; Krivoruchko and Nielsen, 2015). Yeasts, especially *Saccharomyces cerevisiae*, is known as an eukaryotic model microorganism for biosynthesis of high-value metabolites with complicated structures. Unlike a bacterial model *E. coli, S. cerevisiae* possess redox systems that provide the similar physical and physical environment for functional expression of cytochrome P450s and uridine diphosphate glycosyltransferase (UGTs) from plants and mammals. As a result, the ginsenosides skeletons can modify as enzyme hydroxylation and glycosylation (Pompon et al., 1996; Wang C. et al., 2018). The ginsenoside produced in engineered bacteria are limited to small amount due to its less efficient in precursor supplies for methylerythritol phosphate (MEP) pathway (Li et al., 2016; Yu et al., 2019). *S. cerevisiae* is a superior to *E. coli* in production ginsenosides because of its accumulate the sufficient pools of precursors for mevalonic acid (MVA) pathways (Ren et al., 2017). Moreover, compared with the potential cost of yeast-producing ginsenosides (~US$ 0.5–25 per miligram), the value of ginsenosides extracted from *Panax* plants is relatively expensive with a current price of US$ 25–57 per miligram, depending on purity and type of ginsenosides (Dai et al., 2014; Wei et al., 2015; Li et al., 2019; Wang et al., 2019). Therefore, the development of the yeast-producing ginsenosides is expected to continuously enlarge for creating an alternative approach instead of extraction from plant source. In this review, we summarize the current progress in eukaryote microbes, yeasts, for ginsenoside production.

## Chemical Structure And Classification of Ginsenosides

**Figure 1:**
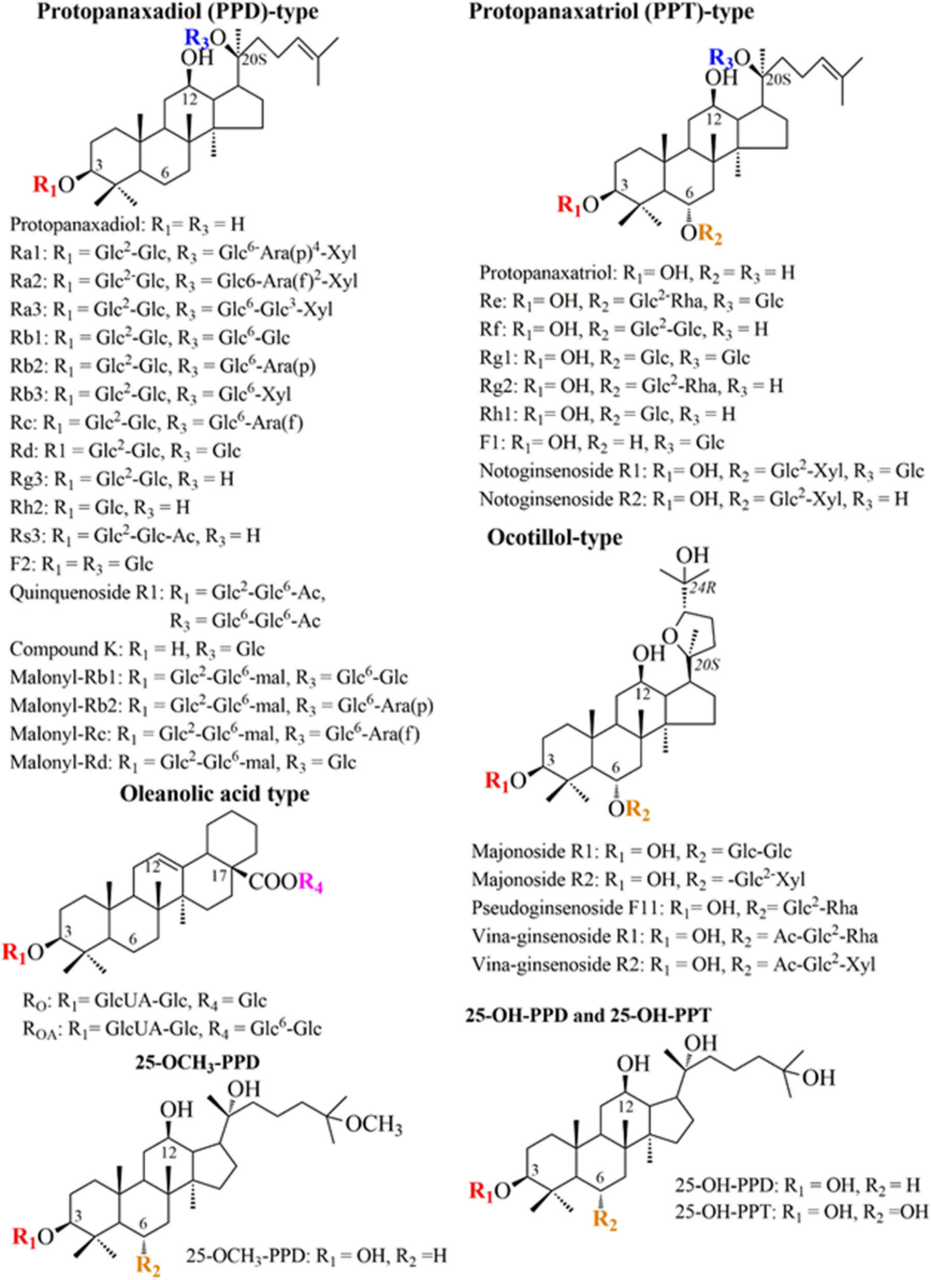
The chemical structures and classification of ginsenosides. Ac, acetyl; Ara(p), α-L-glucopyranosyl; Ara(f), α-L-arabinofuranosyl; Glc, β-D-glucopyranosyl; Rha, α-L-rhamncpyranosyl.

Ginsenosides are steroid-like saponins, which are presented by “Rx.” Wherein “R” indicates the root, and “x” describes the increase in chromatographic polarity based on alphabetic arrangement (Kim et al., 2017). Ginsenosides can be divided into two main groups based on their aglycone skeletons; dammarane-type and oleanane-type (Figure 1). While the structure of dammarane-type ginsenosides includes a tetracyclic ring with sugar moieties, such as glucose, arabinose, xylose, rhamnose, and glucuronic acid, oleanane-type ginsenosides possess pentacyclic skeletons with aglycon oleanolic acid (Shin et al., 2015; Kim et al., 2017). According to carbohydrate moieties attached at the C-3, C-6, and C-20 positions, dammarane-type ginsenosides can be further divided into three subgroups: protopanaxadiol (PPD), protopanaxatriol (PPT), and ocotillol. PPD has a sugar moiety that binds to β-OH at C-3 and/or C-20, including Ra1, Ra2, Ra3, Rb1, Rb2, Rb3, Rc, Rd, Rg3, Rh2, F2, quinquenoside R1, compound K, malony-Rb1, malony-Rb2, malony-Rc, and malony-Rd. The PPT contains Re, Rf, Rg1, Rg2, Rg1, F1, notoginsenoside R1, and notoginsenoside R2, which have a sugar moiety attaching to the α-OH at C-6 and/or β-OH at C-20. The ocotillol group contains majonoside R1, majonoside R2, pseudoginsenoside F11, vina-ginsenoside R1, and vina-ginsenoside R2, which have a five-membered epoxy ring at the C-20 position (Le et al., 2014). PPD and PPT can be found in *P. ginseng* and *P. notoginseng*, whereas the ocotillol group is found in *P. vietnamensis, P. quinquefolius*, and *P. japonicus*. Unlike dammarane-type, oleanane-type ginsenosides, such as Ro and R_OA_, are rare within the *Panax* genus (Wu et al., 2016; Liu et al., 2017; Lin et al., 2019). Recently, new ginsenosides have been isolated and reported, such as 25-OH-PPD, 25-OCH_3_-PPD, and 25-OH-PPT (Figure 1) (Shin et al., 2015; Zhao et al., 2016a). Remarkably, the position and number of -OH groups, the number of sugar moieties, and the stereoselectivity have been reported as the main factors affecting the anticancer activities of ginsenosides (Qi et al., 2010; Nag et al., 2012).

## The Biosynthesis Pathways of Ginsenosides

**Figure 2:**
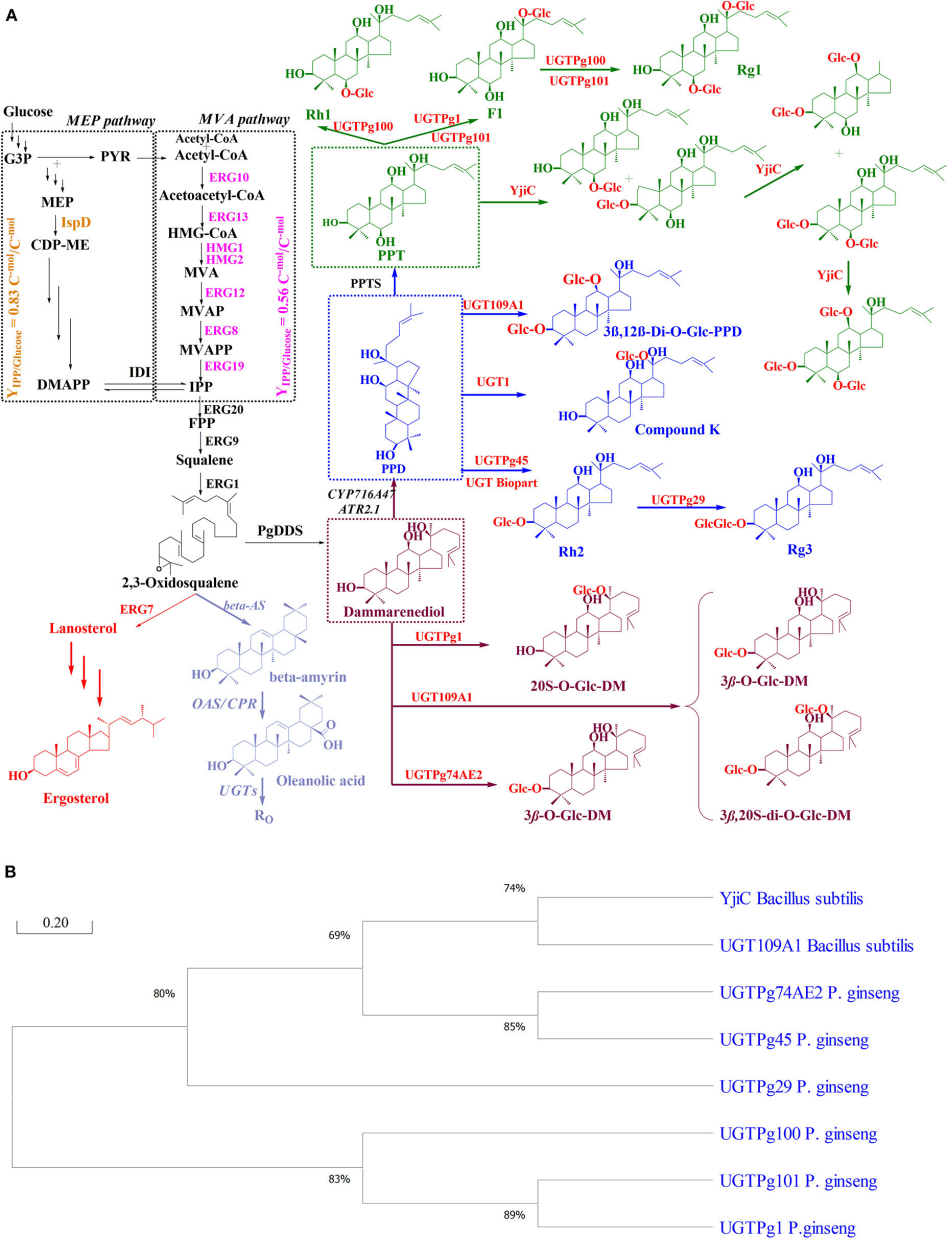
The proposed biosynthetic pathway for ginsenoside production in engineered yeasts. **(A)** Key enzymes and intermediates involved in ginsenoside biosynthesis. ***Enzymes****: b*AS, β*-*amyrin synthase; CPR, cytochrome P450 reductase; CS, cycloartenol synthase; DDS, dammarenediol-II synthase; ERG10, acetyl-CoA C-acetyltransferase; ERG13, HMG-CoA synthase; ERG12, mevalonate kinase; ERG8, phosphomevalonate kinase; ERG19, diphosphomevalonate; ERG20, farnesyl diphosphate synthase; ERG9, squalene synthase; ERG1, squalene epoxidase; ERG7, lanosterol synthase; HMGR, 3-hydroxy-3-methylglutaryl-CoA reductase; IDI, isopentenyl diphosphate-isomerase; IspD, 2-C-methyl-D-erythritol 4-phosphate cytidylyltransferase; OSCs, oxidosqualene cyclases; OAS, oleanolic acid synthase; PPDS, protopanaxadiol synthase; and PPTS, protopanaxatriol synthase. ***Intermediates****:* CDP-ME, 4-Diphosphocytidyl-2-C-methylerythritol; DM, dammarenediol-II; DMAPP, dimethylallyl pyrophosphate; DXP, 1-deoxy-D-xylulose 5-phosphate; FPP, farnesyl diphosphate; GAP, D-glyceraldehyde 3-phosphate; HMBPP, (*E*)-4-Hydroxy-3-methyl-but-2-enyl pyrophosphate; HMG-CoA, 3-hydroxy-3-methylglutaryl coenzyme A; MEP, 2-C-Methyl-D-erythritol-4-phosphate; MVA, mevalonate; MVAP, mevalonate 5-phosphate; MVAPP, mevalonate 5-pyrophosphate; IPP, isopentenyl pyrophosphate; OA, oleanolic acid; PPD, protopanaxadiol; PPT, protopanaxatriol; PYR, pyruvate. **(B)** Phylogenetic analysis of the present UGTs in the biosynthetic pathway. The non-rooted phylogenetic tree was constructed using the neighbor-joining method using MEGA X software. GeneBank accession numbers are for *P. ginseng* UGTPg100 (AKQ76388), UGTPg1 (AIE12479), UGTPg101 (AKQ76389), UGTPg29 (AKA44579), UGTPg74AE2 (AGR44631), UGTPg45 (AKA44586), and *B. subtilis* YjiC (NP_389104), UGT109A1 (ASY97769).

Like terpenoid compounds, ginsenosides are biosynthesized from 2,3-oxidosqualene by utilizing the universal precursors, dimethylallyl diphosphate (DMAPP) and isopentenyl diphosphate (IPP). The MEP and MVA pathways are involved in precursor synthesis and are found in the plastid and cytosol in plant cells, respectively. Notably, it has been reported that ginsenosides are mainly synthesized *via* the MVA pathway in *P. ginseng* (Frank and Groll, 2017; Xu et al., 2017). Moreover, the MEP pathway is present in most prokaryotes and green algae, whereas the MVA pathway is present in most organisms, including animals, yeasts, and archaebacteria (Lombard and Moreira, 2011; Xie et al., 2019). The MEP pathway begins with the formation of –deoxy-D-xylulose–phosphate (DXP) *via* the condensation reaction between D-glyceraldehyde 3-phosphate (GAP) and pyruvate (PYR). Then, the reduction reaction of DXP leads to the formation of 2-C-methyl-D-erythritol-4-phosphate (MEP), followed by several enzymatic steps to convert to (*E*)-4-Hydroxy-3-methyl-but-2-enyl pyrophosphate (HMBPP). Finally, HMBPP is converted to IPP and DMAPP. The MEP pathway requires two ATP and two NADPH to convert one molecule of glucose to one IPP with a yield of 0.833 (Y_IPP/glucose_ = 0.833 C^−mol^/C^−mol^). The condensation from two molecules of acetyl-CoA is the initial step to form IPP and DMAPP through the intermediate mevalonate in the MVA pathway. This pathway takes in 1.5 molecules of glucose and generates one IPP and three NAD(P)H with a yield of 0.56 (Y_IPP/glucose_ = 0.56 C^−mol^/C^−mol^). Notably, isopentenyl diphosphate isomerase (IDI) can catalyze the interconvert reaction between IPP and DMAPP (Figure 2A) (Wang C. et al., 2018).

Subsequently, the condensation of IPP and DMAPP was catalyzed by farnesyl diphosphate synthase (FPS/ERG20) to form farnesyl diphosphate (FPP). Next, FPP was converted into 2,3-oxidosqualene through two enzymatic reaction by squalene synthase (SqS/ERG9) and squalene epoxidase (SqE/ERG1) (Lu et al., 2016). Additionally, 2,3-oxidosqualene is not only a precursor for ginsenosides but also an intermediate substance for phytosterol in plants. The various structures of these compounds are due to the multi-enzymatic reactions, including cyclization, oxidation, and glycosylation (Sun et al., 2019). In *P. ginseng*, cyclization is catalyzed by oxidosqualene cyclases (OSCs) as cycloartenol synthase (CS), β-amyrin synthase (*b*AS), and dammarenediol synthase (DDS). CS is responsible for cycloartenol formation in phytosterol biosynthesis. *b*AS and DDS are catalysts for the conversion from 2,3-oxidosqualene into dammarenediol-II (DM) and β-amyrin in the biosynthesis of dammarane- and oleanane-type ginsenosides, respectively (Liang and Zhao, 2008; Shin et al., 2015). Subsequently, ginsenoside and its derivatives were formed from DM and β-amyrin by tailoring enzymatic reactions. The tailoring enzymes involve cytochrome P450, glycosyltransferase, and acyltransferase (Figure 2A) (Wang C. et al., 2018). Modification reactions not only improve the stability and diversify the structures of ginsenosides but also affect the physicochemical and physiological properties of these compounds (Chu et al., 2016a,b, 2017; Shin et al., 2016).

## Metabolic Engineering of Yeasts For Ginsenosides Production

Yeasts are one of the best hosts for ginsenoside synthesis through metabolic engineering and synthetic biology systems because they are potentially less harmful microorganisms with a high tolerance to plant-derived heterologous enzymes. Importantly, similar intracellular structures to plant cells and the inherent MVA pathway in yeasts produce a sufficient pool of significant precursors for ginsenoside synthesis, such as IPP and DMAPP (Wang C. et al., 2018; Wang et al., 2019). Three yeasts (*Saccharomyces cerevisiae, Yarrowia lipolytica*, and *Pichia pastoris*) have been used as ubiquitous hosts for ginsenoside production. *S. cerevisiae* is a model microbe for the production of various metabolites, while *Y. lipolytica* and *P. pastoris* are unconventional alternatives to *S. cerevisiae*. In this review, we describe the various metabolic engineering strategies that have been developed for improving ginsenoside biosynthesis in yeasts.

### Heterologous Gene Expression

#### Expression of Heterologous Genes From Plants and Microbes

Expression of the heterologous genes from plants or microbes in yeasts provides a possibility for the overproduction of ginsenosides. Three types of enzymes, including glycosyltransferases (GTs), cytochrome P450s (CYP450s), and oxidosqualene cyclases (OSCs), were identified as key enzymes for ginsenoside biosynthesis in *P. ginseng* (Seki et al., 2015; Sun et al., 2019). Therefore, the establishment of the heterologous biosynthetic pathway engineered in yeasts is the first target for enriching the number and diversification of ginsenosides. Among the three enzymes, OSCs control the first step for the formation of diverse cyclic ginsenoside skeletons. Approximately 96 *OSC* genes have been found in higher plants, such as *Arabidopsis thaliana, Sorghum bicolor, Solanum lycopersicum*, and *Oryza sativa* (Wang et al., 2011; Xue et al., 2012). OSCs catalyze 2,3-oxidosqualene to form either protosteryl cations by a chair-boat-chair conformation or dammarenyl cations by a chair-chair-chair conformation, leading to finally forming DM and α/β-amyrin, respectively (Sun et al., 2019). In total, more than 80 *OSC* genes have been cloned and expressed in *S. cerevisiae* and *P. pastoris* (Husselstein et al., 2001; Sun et al., 2019). For instance, the heterologous expression of a new *OSC* gene (named *PNA*), which was obtained from the *P. ginseng* hairy root in a lanosterol synthase deficient *S. cerevisiae* strain GIL77, resulted in DM production (Tansakul et al., 2006). Similar to higher plants, the catalysis of OSCs as *b*AS and DDS in *P. ginseng* was followed by CYP450s and UGTs. CYP450s are monooxygenases that can catalyze various enzymatic reactions, such as hydroxylation, oxidation, and epoxidation (Bernhardt, 2006). CYP450s are present in microbes, plants, and animals for the modification of diverse metabolites with chemo-, stereo-, and regio-selective hydroxylation activity (Chu et al., 2016b; Zhao et al., 2019). Although 414 *CYP450* genes have been identified in *P. ginseng*, only nine genes, including *CYP716A53v2, CYP716A52v2*, and *CYP716A47*, have been functionally verified to contribute to ginsenoside biosynthesis (Wang Y. et al., 2018; Zhao et al., 2019). An enzymatic assay revealed that CYP716A47 catalyzes and converts DM to PPD as dammarenediol 12-hydroxylase, while CYP716A53v2 catalyzes the hydroxylation of PDD at the C-6 position to synthesize PPD (Han et al., 2011, 2012). For PPD production in *S. cerevisiae*, a PPD biosynthetic pathway was constructed through the expression of DDS and PPD synthase from *P. ginseng* along with the NADPH-CYP450 reductase of *A. thaliana* (*At*CPR1). The gene expression cassette initially produced 0.05 mg g/L dry cell weight (DCW) of PPD (Dai et al., 2013). Moreover, the engineered *S. cerevisiae* harboring the PPD biosynthetic pathway could obtain PPT by adding the PPT synthase from *P. ginseng* (Dai et al., 2014). PPD and PPT are ubiquitous aglycons of ginsenosides which undergo glycosylation reactions to form the final ginsenosides (Figure 2A).

Glycosylation is catalyzed by UDP-glycosyltransferase (UGTs), which transfers a sugar moiety from a UDP sugar donor to the hydroxyl groups of ginsenosides. The glycosylation reaction has a significant impact on the solubility, stability, and biological activity of ginsenosides (Pandey et al., 2018; Thuan et al., 2018). A large number of *UGTs* are present in plants, such as *A. thaliana, Glycine max, P. ginseng*, and *Medicago truncatula* (Rahimi et al., 2019). In particular, genome analyses have identified 225 and 242 *UGT* genes in *P. ginseng* and *P. notoginseng*, respectively (Luo et al., 2011; Xu et al., 2017). In addition, novel *UGTs* have been reported in bacteria, such as *Bacillus cereus* 10987 (Hyung et al., 2006), *B. licheniformis* DSM-13 (Pandey et al., 2014), and *B. subtilis* CGMCC 1318 (Liang et al., 2017). Notably, a large number of UGTs have been identified in the biosynthesis of dammarane-type, while UGTs that are responsible for the biosynthesis of oleanane-type are rarely discovered (Seki et al., 2015). Among these UGTs, several functional UGTs from *P. ginseng* and microbes have been analyzed their phylogenetic character (Figure 2B). Because glycosylation is the final enzyme reaction in ginsenoside biosynthesis, it has a significant effect on the diversity of ginsenosides. Therefore, the introduction of *UGTs* into PPD/PPT-producing engineered yeast can result in the biosynthesis of ginsenosides and their analogs. For instance, 240 μg/L of compound K can be obtained by expressing *P. ginseng UGTPg1* in the PPD-producing chassis strain of *S. cerevisiae*. This research also demonstrated that *P. ginseng* UGTPg1 could catalyze the conversion of Rg3 and Rh2 to F2 and Rd, respectively (Yan et al., 2014). In another study, the characterization of two UGTs from *P. ginseng* indicated that UGTPg45 could transfer the glucose moiety to the hydroxyl group at C-3 of PPD to form Rh2. UGTPg29 can link the 1-2 glycosidic bond at the hydroxyl group of C-3 glucose of Rh2, resulting in Rh3 production. The integration of *UGTPg45* or both genes (*UGTPg45* and *UGTPg29*) into the δ*-*DNA site of PPD-producing chassis strain of *S. cerevisiae* resulted in the production of Rh2 and Rh3. The yield of Rh2 and Rh3 was achieved in the engineered strain with 1.45 and 3.49 μmol g/L DCW, respectively (Wang et al., 2015). Subsequently, PPT and its derivatives were biosynthesized in engineered yeast. For example, the biosynthesis of Rh1 and F1 was achieved by introducing *P*. *ginseng UGTPg1* and *UGTPg100* into PPT-producing engineered *S. cerevisiae*. UGTPg1 and UGTPg100 were able to specifically catalyze the glycosylation at the hydroxyl group of C-20 and C-6 of PPT, respectively, and produce Rh1 and F1 with a titer of 92.8 and 42.1 mg/L, respectively (Table 1; Figure 2) (Wei et al., 2015). These examples indicate that the construction of a heterologous pathway, as well as the *de novo* biosynthetic pathway, have a significant role in ginsenoside production in yeasts. However, the product yield still requires improvement through synthetic biology and metabolic engineering, as well as a systems approach.

**Table 1:** Summary of ginsenoside production in engineered yeasts.

**Strains**	**Related gene cassettes in biosynthesis pathway**	**Type of ginsenosides**	**Cultivation condition**	**Major media**	**Carbon source**	**Titer (mg/L)**	**References**
***Saccharomyces cerevisiae***							
ZD-PPD-018	P_PGK1_-*tHMG1*-T_ADH1_, P_TDH3_-*AtCPR1*-T_TPI1_, P_TEF1_-*SynPgPPDS*-T_CYC1_, P_PGK1_-*ERG20*-T_ADH1_, P_TDH3_-*ERG1*-T_TPI1_, P_TEF1_-*ERG9*-T_CYC1_	PPD DM	Fed-batch	SD	Glucose	1,189 1,548	Dai et al., 2013
GY-1	P_PGK1_-*GgbAS*-T_ADH1_, P_TDH3_-*AtCPR1*-T_TPI1_, P_TEF1_-*MtOAS*-T_CYC1_, P_PGK1_-*PgDDS*-T_ADH1_, P_FBA1_-*SynPgPPTS*-T_TDH2_, P_TDH3_-*AtCPR1*- T_TPI1_, P_TEF1_-*SynPPDS*-T_CYC1_	PPD PPT Oleanolic acid	Shake-flask	YPD	Glucose	17.2 15.9 21.4	Dai et al., 2014
D20RH18	P_TEF1_-*PgDDS*-P_CYC1_, P_TEF1_-*synPgPPD*S-T_FBA1_, P_TDH3_-*ATR2.1*-T_ENO2_, P_PGK1_-*tHMG1*-T_ADH1_, P_GPM1_-*ERG20*-T_CYC1_, P_TPI1_-*PgERG1*-T_ENO2_, P_PGK1_-*ERG9*-T_FBA1_, P_TEF1_-*UGTPg45*-T_CYC1_	Rh2	Shake-flask	YPD	Glucose	1.45 μmol/g DCW	Wang et al., 2015
D20RG1	P_TEF1_-*PgDDS*-P_CYC1_, P_TEF1_-*synPgPPDS*-T_FBA1_, P_TDH3_-*ATR2.1*-T_ENO2_, P_PGK1_-*tHMG1*-T_ADH1_, P_GPM1_-*ERG20*-T_CYC1_, P_TPI1_-*PgERG1*-T_ENO2_, P_PGK1_-*ERG9*-T_FBA1_, P_TEF1_-*UGTPg45*-T_CYC1_, P_HXT7_-*UGTPg29*-T_CYC1_	Rh3				3.49 μmol/g DCW	
ZW-Rh1-20	P_GPM1_-*ERG20*-T_CYC1_, P_TPI1_-*PgERG1*-T_ENO2_, P_PGK1_-*ERG9*-T_FBA1_, P_PGK1_-*tHMG1*-TADH1, P_TEF1_-*CYP716A53v2*-T_FBA1_, P_TDH3_-*PgCPR1*- T_ADH1_, P_PGK1_-*UGTPg100*-T_TPI1_	Rh1 PPT PPD DM	Shake-flask	SC	Glucose	98.2 3.5 43.4 8.8	Wei et al., 2015
ZW-F1-17	P_GPM1_-*ERG20*-T_CYC1_, P_TPI1_-*PgERG1*-T_ENO2_, P_PGK1_-*ERG9*-T_FBA1_, P_PGK1_-*tHMG1*-TADH1, P_TEF1_-*CYP716A53v2*-T_FBA1_, P_TDH3_-*PgCPR1*- T_ADH1_, P_PGK1_-*UGTPg1*-T_TPI1_	F1 PPT CK PPD DM			Ethanol	42.1 13.9 7.5 49.2 3.5 138.8	
ZY-M7(4)EΔ-PUA	P_PGK1_-*ERG20*-T_ADH1_, P_TDH3_-*ERG1*-T_TPI1_, P_TEF1_-*ERG9*-T_CYC1_, P_PGK1_-*tHMG1*-T_ADH1_, P_TDH3_-*M7-1*-T_CYC1_, *ΔEGH1*, P_PGK1_-*PGM1*, P_HX17_-*UGP1*, P_TEF1_-*PgPPDS-AtCPR2*-T_CYC1_	Rh2	Fed-batch	SC	Glucose	300	Zhuang et al., 2017
IN-B	P_GAL10_-*DS-GFP*-T_ADH1_, P_GAL1_-*ATR1*-T_CYC1_, P_GAL1_-*PPDS*-T_CYC1_; P_GAL10_-*ERG7*-T_ADH1_, P_GAL1_-*tHMG1*-T_CYC1_; P_GAL10_-*UGT109A1*-T_ADH1_	3β,12β-Di-O-Glc-PPD DM PPD	Shake-flask	SG	Galactose	9.05 4.57 11.5	Liang et al., 2017
W3a	*DS, ERG1, tHMG1, ERG20, ERG9, PPDS-*linker1*-*46*tATR1*	PPD	Fed-batch	YPD	Glucose	1436.6	Zhao et al., 2016c
W3a-ssPy	*DS, ERG1, tHMG1, ERG20, ERG9, PPDS-*linker1*-*46*tATR1*, P_PGK1_-*YBP1*-T_CYC1_, P_GK1p_-*SSD1*-T_ADH1_	PPD	Fed-batch	YPD	Glucose, ethanol	4,250	Zhao et al., 2017
WLT-MVA5	P_ALD6_-*DS*-T_CYC1_, P_TDH3_-*PPDS-ATR1*-T_ADH3_, P_TEF1_-*ERG1*-T_ADH1_, P_TEF1_-*tHMG1*-T_CYC1_, P_ACS1_-*ERG9*-T_ADH2_, P_TEF2_-*ERG20*-T_ADH4_, P_TEF2_-*ERG10*, P_MLS1_-*ERG13*, P_ALD6_-*ERG12*, P_SSA1_-*ERG8*, P_CIT2_-*ERG19*, P_IDP2_-*IDI1*, P_PGK1_-*NCP1*, P_ACS1_-*TetR*-T_ADH2_, P_TEF1_-*ACS_*seL*641*P*_*-T_ADH1_	PPD	Fed-batch	YNBD	Glucose/ Ethanol	8,090	Zhao F. L. et al., 2018
PPD08	P_TEF1_-*ERG20*, P_CCW12_-*tHMG1*, P_GPD_-*AtCPR1*, P_ADH2_-*PgDS*, P_ADH2_-*PgPPDS*, P_GPD_-*ald6*	PPD	Shake-flask	YSC	Glucose	6.01	Kim J. E. et al., 2018
Y1CSH	P_TEF1_-*HAC1*-T_CYC1_, P_TDH3_-*IDI1*-T_TPI1_, P_PGK1_-*ERG20*-T_ADH1_, P_TEF1_-*ERG9*-T_CYC1_, P_PGK1_-*ERG1*-T_ADH1_, P_TEF1_-*ERG7*-T_CYC1_, P_TEF1_-*synDS-GFP*-T_CYC1_, P_PGK1_-*tHMG1*-T_ADH1_, P_TDH3_-*synPgUGT74AE2*-T_ADH2_	3β-O-Glc-DM	Fed-batch	YPD	Glucose	2,400	Hu et al., 2019
Y2CSH	P_TEF1_-*HAC1*-T_CYC1_, P_TDH3_-*IDI1*-T_TPI1_, P_PGK1_-*ERG20*-T_ADH1_, P_TEF1_-*ERG9*-T_CYC1_, P_PGK1_-*ERG1*-T_ADH1_,	20S-O-Glc-DM				5,600	
	P_TEF1_-*ERG7*—T_CYC1_, P_TEF1_-*synDS-GFP*-T_CYC1_, P_PGK1_-*tHMG1*-T_ADH1_, P_TDH3_-*synUGTPg1*-T_ADH2_						
ZWDRH2-10	P_HXT7_-*tHMG1*-T_ADH1_, P_TEF2_-*synPgCPR1*-T_TDH2_, P_TPII_-*ERG1*-T_ENO2_, P_GPM1_-*ERG20*-T_CYC1_, P_PGK1_-*ERG9*-T_FBA1_, P_TDH3_-*synDDS*-T_PGT1_, P_TEF1_-*synPPDS*-T_PGK1_, P_ENO2_-*ERG12*-T_CPS1_, P_TEF2_-*ERG13*-T_IDP1_, P_TPII_-*ERG8*-T_PRM5_, P_GPM1_-*ERG19*-T_HIS5_, P_PGK1_-*IDI-*T_PRM9_, P_TDH3_-*ERG10*-T_SPG5_, P_TEF1_-*tHMG1*-T_ADH1_, P_TDH3_-*UGTPn50-HV*-T_PRM9_	Rh2 PPD DM	Fed-batch	YPD	Glucose	2,252.3 9,054.5 8,088.8	Wang et al., 2019
***Yarrowia lipolytica***							
Y14	*Ku70* deletion::*LUL*, P_EXP1P_-*XYL1*-T_XPR2_, P_GPD1_-*XYL2*-T_LIP2_, P_TEF1_-*ylXKS*-T_CYC1_, P_TEF1_-*DS*-T_XPR2_, P_EXP1_-*PPDS-*linker-*ATR1*-T_LIP2_, P_FBAIN_-*tHMG1*-T_XPR2_, P_GPD1_-*ERG9*-T_CYC1_, P_EXP1_-*ERG20*-T_LIP2_, P_EXP1_*-TKL*-T_MIG1_, P_TEF1_-*TAL*-T_LIP2_, P_FBA1_-*TX*-T_CYC1_	PPD	Fed-batch	YPD or YPX	Xylose Glucose	300.63 167.17	Wu et al., 2019
YL-MVA-CK	P_FBAIN_-*tHMG1*-T_XPR2_, P_EXP1_-*ERG9*-T_MIG2_, P_GPD1_-*ERG20*-T_CYC1_, P_TEF1_-*OpDS*-T_XPR2_, P_EXP1_-*PPDS*-*linker2-ATR1*-T_MIG1_, P_GPD1_-*UGT1-*T_LIP2_	Compound K	Fed-batch	YPD	Glucose	161.8	Li et al., 2019
***Pichia pastoris***							
KDPEP	*PgDDS*-L3-PDZlig and *ERG1*-ER/kPDZ with p-[*PgDDS*-PDZlig]/[*ERG1*-PDZ]	DM	Shake-flask	YPD	Glucose, methanol	0.10 mg/g DCW	Zhao et al., 2016b

*SynPg, genes from Panax ginseng with codon optimization; P, promoter; T, Terminator*.

#### The Modular Optimization Strategy

Because multiple genes with different sources are included in the ginsenoside biosynthetic pathway in yeasts, optimal metabolic pathway strategies are required for high product yield. In general, there are four modules in the biosynthesis pathway for the production of ginsenosides. The first module produces acetyl-CoA from a carbon source, such as glucose or ethanol, by overproduction of *ZWF, ALD6*, and *ACS* (Kim J. E. et al., 2018; Zhao F. L. et al., 2018). Recently, the first novel module was found to contain genes encoding xylose reductase (XR) and xylitol dehydrogenase (XDH) for acetyl-CoA synthesis from xylose in *Y. lipolytica* (Wu et al., 2019). The second module is the MVA pathway module, which converts acetyl-CoA to IPP/DMAPP. The genes encoding ERG10, ERG13, tHMG, ERG12, ERG8, ERG19, and IDI belong to the second module. The third module contains the genes encoding ERG20, ERG9, and ERG1 for increasing the availability of 2,3-oxidosqualene from IPP/DMAPP. The last module contains genes encoding three key heterologous enzymes (PgDDS, PgCPR1, or PgPPDS and UGTs) for ginsenoside production. The optimization of genetic cassettes is related to the combination of different genetic elements, including promoter strength, synthetic transcription factors, the terminator region of each gene, and the copy number. Each genetic cassette was assembled as promoter-gene-terminator. A few general terminators were amplified from the yeast genome and used for ginsenoside production, such as *T*_*ADH*1_, *T*_*TPI*1_, *T*_*CYC*1_, *T*_*FBA*1_, *T*_*TYS*1_, *T*_*GPM*1_, and *T*_*ALA*1_ (Yamanishi et al., 2013; Zhang G. et al., 2015). The major promoter used in ginsenoside production is the constitutive promoter, which is found in the glycolytic pathway of *S. cerevisiae*. Among the vast native promoters, promoter *P*_*TEF*1_ has the highest strength under the available glucose conditions, followed by promoters *P*_*PGK*1_, *P*_*TDH*3_, *P*_*TP*__*I*1_, *P*_*PYK*1_, and *P*_*ADH*1_, while *P*_*HXT*7_ has the weakest strength (Partow et al., 2010). On the contrary, *P*_*HXT*7_ has a higher strength than the other promoters of *P*_*PGK*1_, *P*_*TPI*1_, *P*_*TDH*3_, *P*_*PYK*1_, and *P*_*ADH*1_ in glucose-limited conditions and the presence of ethanol (Partow et al., 2010; Sun et al., 2012). Recently, the native constitutive promoters for enhancing the target product have replaced strong artificial promoters. For example, the 3-fold enhancement of the Rh2 yield was achieved using the strong artificial UAS-TDH3 promoter for controlling UGTPg45-HV (Wang et al., 2019). Notably, the overexpression of each module could be achieved by using either a plasmid system with a different copy number or integration into the host genome (Zhou et al., 2012; Redden et al., 2015). In general, increasing copy numbers of the genes involved in the rate-limiting enzymes or ginsenoside biosynthesis in engineered yeast will improve the target compound production. However, the highest copy number of the genes did not always lead to the highest product yield due to the metabolic burden in engineered yeast (Hu et al., 2019; Li et al., 2019). The integration of exogenous DNA into the yeast genome can be obtained through homology-direct recombination (HDR) or non-homologous end joining (NHEJ). For example, clustered regularly interspaced short palindromic repeats-CRISPR associated 9 nuclease (CRISPR-Cas9) DHR has been used to integrate multi-copy *DDS* and *UGT* genes to improve 3β-*O*-Glc-DM and 20*S*-*O*-Glc-DM production in *S. cerevisiae* (Hu et al., 2019). To increase the rate of HDR from 28 to 54%, block NHEJ was performed by the knockout *Ku70* cassette in *Y. lipolytica* (Wu et al., 2019).

### Balancing and Increasing Metabolic Flux

The introduction of the heterologous pathway in yeast is the most important part of metabolic engineering for ginsenoside biosynthesis. This approach might cause an unbalanced cellular metabolic flux. The competitive consumption of the precursor metabolite, cofactor, and substrate supplied between the endogenous and heterologous pathways not only results in a reduction of target compound productivity but also affects cell growth (Gupta et al., 2017). Therefore, balancing and increasing these factors would facilitate the enhancement of ginsenoside production in recombinant yeasts.

### Balancing and Increasing Precursor Pools

**Figure 3:**
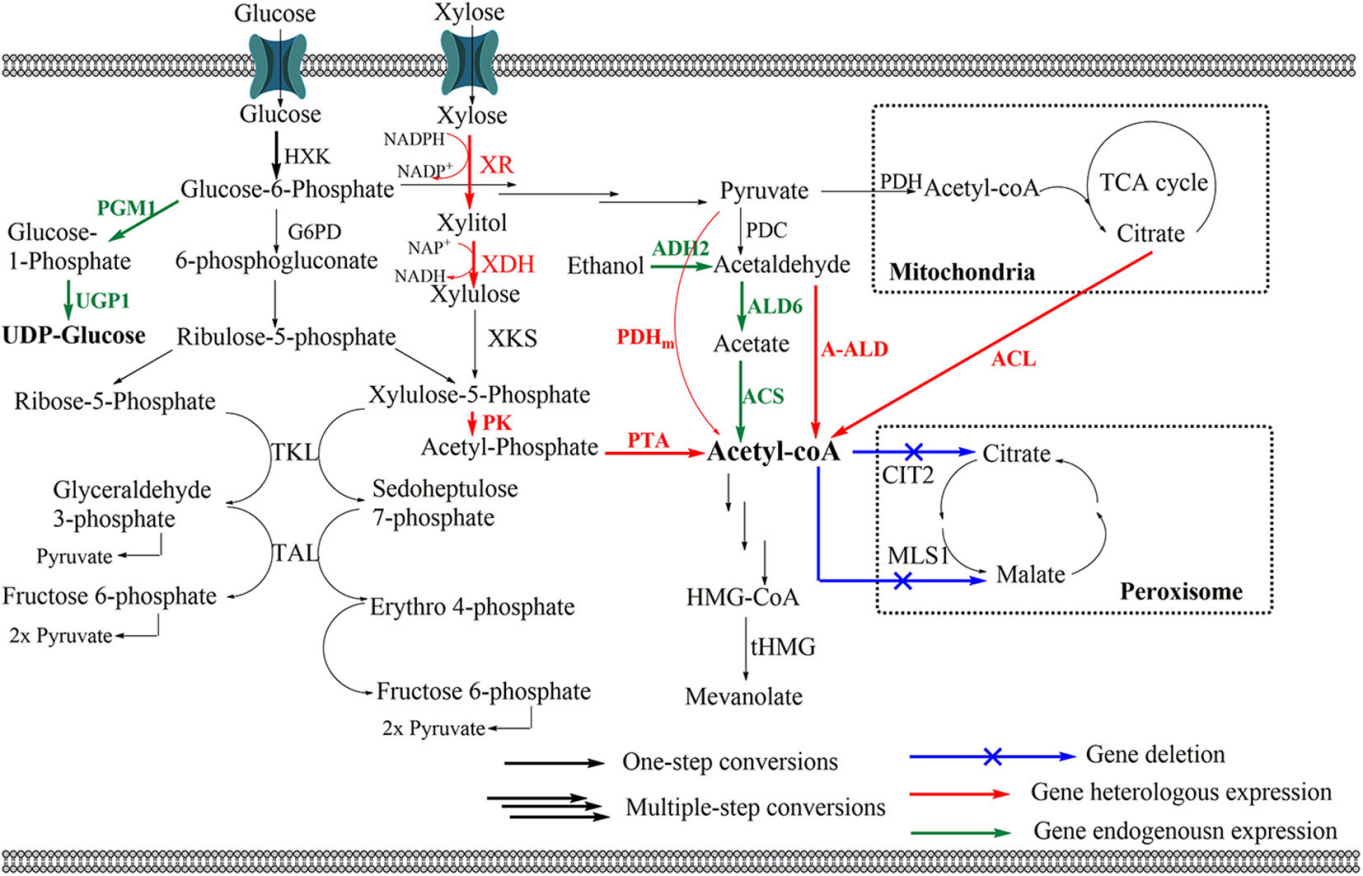
Biosynthetic pathway for acetyl-CoA in yeast. ***Enzymes****:* 6PGDH, 6-phosphogluconate dehydrogenase (encoded by *gnd1*); ACL, ATP citrate lyase from *Aspergillus nidulans*; ACS, acetyl-CoA synthase; ALD2 and ALD6, acetaldehyde dehydrogenases, encoding by *ald2* and *ald6*, respectively; A-ALD, acetylating acetaldehyde dehydrogenase from *E. coli*; CIT2, citrate synthase; G6PD, glucose 6-phosphate dehydrogenase (encoded by *zwf1*); GDH, glutamate dehydrogenase; HXK, hexokinase (encoded by *hxk1*); MLS1, malate synthase; PDH, pyruvate dehydrogenase complex from *E. coli*; PDHm, modified NADP-dependent PDH; PK, phosphoketolase from *Leuconostoc mesenteroides*; PTA, phosphotransacetylase from *Clostridium kluyveri*; PGM, phosphoglucomutase (encoded by *pgm1*); TAL, transaldolase; TKL, transketolase; UGP, glucose-1 phosphate uridylyltransferase (encoding by *ugp1*); XDH, xylitol dehydrogenase; XKS, Xylulose kinase; XR, xylose reductase. *Intermediates:* E-4-P, erythrose-4-phosphate; F6P, Fructose 6-phosphate; G-3-P, glyceraldehyde-3-phosphate; HMG-CoA, hydroxymethylglutaryl-CoA; MVA, mevalonate acid; PPP, pentose phosphate pathway.

In the ginsenoside biosynthetic pathway, acetyl-CoA is the starting precursor for the formation of IPP/DMAPP in the MVA pathway of yeasts (Pronk et al., 1996; Chen et al., 2013). However, a difficulty in overproducing ginsenosides is the lack of intracellular acetyl-CoA levels in the host cell. Therefore, the most effective strategy for this challenge is promoting the supply of acetyl-CoA. First, an effective approach is to make the endogenous gene more active (Chen et al., 2012, 2013; Kocharin et al., 2012; Kozak et al., 2014). For example, *ALD6* (encoding NADP-dependent aldehyde dehydrogenase) overexpression along with the introduction of a synthetic codon-optimized acetyl-CoA synthase mutant from *Salmonella enterica* (*ACSse*_*L*641*P*_) shown to increase cytoplasmic acetyl-CoA, resulted in an improvement of PPD production. The engineered *S. cerevisiae* WLT-MVA5 produced up to 66.55 mg/L (OD_600_ = 6.18) of PPD (Zhao F. L. et al., 2018). Using the same strategy, increasing the cytoplasmic acetyl-CoA supply in *Y. lipolytica* is possible through the overexpression of endogenous transaldolase (*TAL*) and transketolase (*TKL*). As a result, the PPD title was improved by up to 88.73 mg/L using xylose as a carbon source (Wu et al., 2019). Furthermore, it has been demonstrated that the engineering of the pyruvate dehydrogenase (PDH) bypass, ATP-dependent citrate lyase (ACL) route, and the PDH pathway also resulted in enhanced intracellular acetyl-CoA in yeast (Figure 3) (Lian et al., 2014; Krivoruchko et al., 2015; Huang et al., 2018). Interestingly, a xylulose 5-phosphate-specific phosphoketolase (*PK*) and phosphotransacetylase (*PTA*) were expressed along with acetylating acetaldehyde dehydrogenase (*A-ALD*), resulting in acetyl-CoA level enhancement (van Rossum et al., 2016). The various approaches of engineering acetyl-CoA metabolism provided an efficient strategy for the enhancement of ginsenoside biosynthesis.

Since 3-hydroxy-3-methylglutaryl-coenzyme A (HMG) reductase (HMG1, HMG2) showed the activity as the post-transcriptional feedback inhibition in the MVA pathway, the approaches for improving the conversion rate from HMG to MVA were considered for ginsenoside production (Polakowski et al., 1998). One way to tackle this is to overexpress the truncated N-terminal of HMG1 (*tHMG1*), which lacks its N-terminal transmembrane sequence coding for membrane-binding activity. As expected, integrating *tHMG1* under the control of *P*_*PGK*1_ promoter into the δ-DNA site of the PPD-producing strain led to the improvement of at least 89.8-fold for the PPD yield with 4.49 mg/g DCW (Dai et al., 2013). Furthermore, the overexpression of *tHMG1* along with a global transcriptional element of sterol biosynthesis (*UPC2*) is an effective approach to increase MVA flux (Dai et al., 2012, 2013). As expected, the overexpression of *tHMG1* along with *UPC2* in compound K-producing *S. cerevisiae* resulted in compound K production from glucose and galactose with 0.8 and 1.4 mg/L, respectively (Yan et al., 2014). In addition, improving the conversion rate of intermediate molecules is one of the important approaches to enhancing ginsenoside production. The intermediate molecules include FPP, squalene, and 2,3-oxidosqualene. Three genes (*ERG20, ERG9*, and *ERG1* from *S. cerevisiae* BY4742) were integrated into ginsenoside-producing yeast along with *tHMG1*. As a result, the biosynthesis of DM and PPD was enhanced by up to 10.9- and 1.9-fold with 10.97 and 8.48 mg/g DCW, respectively (Dai et al., 2013). Recently, overexpression of the *IDI* gene accompanied expression of other genes in the MVA pathway to improve the production of 3β-*O*-Glc-DM and 20S-*O*-Glc-DM in the engineered *S. cerevisiae* (Figure 3) (Hu et al., 2019).

#### Engineering the Cofactor Level

It is important to not only regulate the presence of the precursor but also enhance the availability of the cofactor level. The redox enzymes, including tHMG1, ERG9, CYP450s, and PgPPD synthase, use NADPH as a major cofactor (Dai et al., 2012, 2013; Yan et al., 2014). It has been reported that the cytosolic NADPH concentration can be increased through either the overexpression of genes encoding NADPH-generating enzymes or the deletion/inhibition of genes encoding NADPH-utilizing enzymes. These genes are presented in the pentose phosphate pathway (PPP), glutamate dehydrogenase (GDH) pathway, and acetate pathway of yeast (Hector et al., 2011). In the PPP, glucose 6-phosphate dehydrogenase (G6PH, ZWF1) catalyzes the conversion of glucose 6-phosphate to 6-phospho gluconolactone, and 6-phosphogluconate dehydrogenase (6PGDH, GND1) converts 6-phospho gluconate to ribulose-5-phosphate. Oxidation by both enzymes is carried out along with the reduction of NADP^+^ to NADPH, which is considered the major factor modulating the reductive biosynthetic reaction in yeast (Hector et al., 2011).

Moreover, it has been identified that the *STB5* gene encoding a transcription factor is a basal regulator of PPP. Although the overexpression of *ZFW1* or *STB5* increased the intracellular NADPH level by 1.4- and 1.2-fold, respectively, it also led to a decrease in PPD production. Overexpression of these genes has been demonstrated to compete and repress the glycolysis pathway for accumulating acetyl-CoA, and cause an unbalanced carbon flux in PPD production (Kim J. E. et al., 2018). In the GDH pathway, GDH1 catalyzes the formation of glutamate from α-ketoglutarate through the reductive amination reaction using NADPH as the donor proton. In contrast, GDH2 is a NAD-dependent isozyme and is responsible for the oxidative deamination of glutamate (Ljungdahl and Daignan-Fornier, 2012). Deletion of *GDH1* coupled with *GDH2* overexpression led to increased intracellular NADPH concentration by 1.5-fold. However, PPD production was not effective in compare with parent strain. However, PPD production was not increased (Kim J. E. et al., 2018). Importantly, the enhancement of NADPH availability, along with the improvement of the NADPH/NADP^+^ ratio, increased PPD production. The overexpression of *ALD6* in place of *ALD2* led to a 3-fold increment of the cytosolic NADPH/NADP^+^ ratio. As a result, the PPD production of the engineered strain obtained was 6.01 mg/L, which was an 11-fold improvement on the titer of PPD in comparison with parent strains (Kim J. E. et al., 2018). Both cytoplasmic acetaldehyde dehydrogenases encoded by *ALD2* and *ALD6* catalyze the conversion of acetaldehyde to acetate. While ALD2 uses NAD^+^ as the preferred cofactor, ALD6 uses NADP^+^ as the preferred cofactor (Minard and McAlister-Henn, 2005). These results indicate that the improvement of ginsenoside production is highly correlated with the available NADPH and NADPH/NADP^+^ ratio.

UDP sugar has been used as a donor sugar in glycosylation reactions in ginsenoside production. The supply of UDP-sugar as UDP-glucose is necessary because it is one of the major limiting factors for efficient ginsenoside biosynthesis in yeast. Among three endogenous genes (*HXK1* encoding hexokinase, *PGM1* encoding phosphoglucomutase, and *UGP1* encoding glucose-1 phosphate uridylyltransferase), PGM1 and UGP1 are involved in the rate-limiting steps in the UDP-glucose biosynthetic pathway from glucose. PGM1 catalyzes the conversion of glucose-6-phosphate to glucose-1-phosphate, then glucose-1-phosphate is converted to UDP-glucose by UGP1 (Figure 3) (Hou et al., 2017). For example, the overexpression of *PGM1* under the control of the P_PGK1_ promoter along with *UGP1* under the control of the P_HXT7_ promoter was conducted to produce Rh2 in *S. cerevisiae* strains. As a result, the engineered strain ZYM7 (4)-E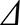-PU produced 65% more Rh2 than the parent strain, ZYM7(4)-E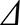, with 36.7 mg/L from glucose (Table 1) (Zhuang et al., 2017).

#### Decreasing the Flux of Branch Pathway

Since increasing the acetyl-CoA is beneficial for improving the overall flux of the MVA pathway in yeasts, the deletion of genes involved in the glycolysis pathway, tricarboxylic acid (TCA) cycle, glyoxylate cycle, and related-amino acid metabolism is considered (Nielsen, 2014; Sun et al., 2014). Acetyl-CoA enters TCA and glyoxylate cycle by citrate synthase (CIT2). Moreover, malate synthase (MLS1) is responsible for the transport of acetyl-CoA into glyoxylate cycle in peroxisome. Therefore, the knock-out of these genes leads to improving the accumulation of acetyl-CoA in the cytoplasm (Figure 3) (Chen et al., 2014; Feng et al., 2015). Interestingly, deletion of genes associated with amino acid metabolism, and fructose and mannose metabolism was reported to the effect on production of DM in engineered *S. cerevisiae* YPH499 and INVSc1. While phosphoglycerate dehydrogenases (SER3) catalyzes the first step of the phosphorylated L-serine biosynthesis pathway, sorbitol dehydrogenase 1 (SOR1) catalyzes the reversible NAD^+^-dependent oxidation of sorbitol (Sun et al., 2014). The cell growth of the mutant rapidly decreased compared to the wild type due to the inactivation of the *SER3* and *SOR1* genes, which have a strong impact on cofactor availability, such as NAD(P)H or NAD(P)^+^. Only the engineered strain, Y-ΔHXK2, harboring the deletion of *HXK2* (encoding for hexokinase 2), was able to grow slightly faster than the control. As a result, the titer of DM from mutant Y-ΔHXK2 was two-fold higher than the wild type with 18.26 mg/L (Hu et al., 2019). This may be because the *HXK2* mutant caused reduced glucose repression and redirected the carbon flux to the MVA pathway for IPP/DMAPP biosynthesis (Diderich et al., 2001).

Precursors, such as squalene and 2,3-oxidosqualene, have been used as intermediate metabolites for sterol biosynthesis (Veen et al., 2003). Therefore, the competitive pathway is repressed, as it is apparent that sterol biosynthesis is a beneficial strategy for improving ginsenoside production in engineered yeast. First, the replacement of the native *ERG7* promoter by the methionine repressible promoter *P*_*MET*3*p*_ was performed to decrease the expression level of *ERG7*, which is necessary for lanosterol and ergosterol. However, methionine induction led to complicate matter during fermentation and increase the product cost. Subsequently, the *ERG7* promotor was modified to identify the approximate expression strength using a Tetracycline repressor-Tetracycline operator (*TetR*-*TerO*) gene regulation system (Hu et al., 2017). As expected, a significant decrease was detected in lanosterol concentration without the effect on cell growth from the WLT-MVA1-EC strain, which was an engineered *S. cerevisiae* strain harboring native *ERG7p* of strain WLT-MVA1 replaced by *ERG7Cp* promoter. Although the ergosterol levels remained the same in this strain, ~15% of PPD production increased with the addition of 45 mg/L (OD_600_ = 7.21). Notably, the WLT-MVA1-EC strain harboring *DDS* under *TEF1p* promoter achieved up to 51.42 mg/L of PPD production (OD_600_ = 7.07) without the detection of 2,3;22,23-oxidosqualene (Zhao F. L. et al., 2018). Secondly, it is a ubiquitous strategy to delete the endogenous genes encoding sterol biosynthesis enzymes by the integration of the genes encoding heterologous enzymes for ginsenoside production. For example, two genes encoding lipid phosphate phosphatase (*LPP1*) and diacylglycerol-pyrophosphate phosphatase (*DPP1*) in *S. cerevisiae* are responsible for farnesol (FOH) biosynthesis from FPP (Scalcinati et al., 2012). In order to improve the flux toward PPD biosynthesis *via* FPP, three genes (*AtCPR1, PgDDS*, and *PgPPDS*) were integrated at the *LPP1, DPP1*, and *YPL062w* sites in the chromosome of *S. cerevisiae*, resulting in the increased production of PPD (0.54 mg/L) at 144 h (Kim J. E. et al., 2018).

### Improving Enzymes for Efficient Biocatalysts

#### Investigating of High Substrate-Flexibility Enzymes From Microbes

The high substrate specificity of plant UGTs is the cause of the limitation of the compound number in ginsenoside biosynthesis. For example, UGRdGT from *P. notoginseng* converts Rd to Rb1 by forming a 20-*O*-glycosidic linkage at the C-20 position (Yue and Zhong, 2005), while PgUGT74AE2 transfers glucose from UDP-glucose to PPD and CK at the C-3 position to produce Rh2 and F2, respectively (Jung et al., 2014). UGT74M1 from *Saponaria vaccaria* showed glycosylation activity at C-28 of gypsogenic acid and quillaic acid to produce oleanane-type ginsenosides (Meesapyodsuk et al., 2007). Soybean UGT73F4 transfers xylose to the arabinose moiety of soyasapogenol A at the C-22 position (Sayama et al., 2012). Therefore, the discovery of novel ginsenosides within the substrate specificities of UGTs requires immediate attention to determine the complete genome sequencing and ginsenoside biosynthetic pathway in a large number of organisms. In addition, the poor solubility and expression levels of enzymes from plants cause the low productivity of target compounds. The screening and characterization of UGTs remain practical challenges. The amplification of the substrate flexibility of UGTs from microorganisms is very important to produce novel ginsenosides in yeast. For example, a bioactive unnatural ginsenoside, such as 3,12-Di-*O*-β-D-glucopyranosyl-dammar-24-ene-3β,12β,20S-triol (3β,12β-Di-O-Glc-PPD), could be obtained by the overexpression of *B. subtilis UGT109A1*, which catalyzes the transfer of glucose moiety to the hydroxyl group at C-3, C-12, and C-20 of ginsenosides in *S. cerevisiae* (Liang et al., 2017). Furthermore, *in vitro* enzymatic reactions demonstrate that a broad UGT substrate from *B*. *subtilis* 168 (YjiC) can catalyze the glycorandomization of PPT at hydroxyl groups C-3, C-6, and C-20 for Rh1 biosynthesis and four unnatural ginsenosides (Pandey et al., 2013; Dai et al., 2018). Moreover, enzymes with substrate flexibility have been characterized, such as the oleandomycin GT and its mutant form *Streptomyces antibioticus*, MhGT1 from *Mucor hiemalis*, and GT1 from *B. cereus* (Gantt et al., 2008; Chiu et al., 2016; Feng et al., 2017). The amplification of these enzymes in recombinant yeasts could further improve the proficiency and novel ginsenoside biosynthesis in whole cells.

#### Enhancing Enzyme Activity

Although the substrate flexibility of UGTs made ginsenoside biosynthesis possible, the catalytic efficiency of the native enzymes remained low. Therefore, enzyme engineering is necessary to significantly improve production. For instance, UDP-glucose sterol glucosyltransferase from *S. cerevisiae* (UGT51) not only catalyzes a native substrate as ergosterol, sitosterol, cholesterol, and pregnenolone to form their glucosides but also converts PPD and PPT to ginsenoside glucosides. The conversion efficiency from PPD to Rh2 in an *in vitro* enzymatic assay was 13% using the native enzyme UGT51. The combination of semi-rational design based on the crystal structure, alanine scanning mutagenesis, and iterative saturation mutagenesis resulted in the generation of the best mutant, M7-1, which includes seven mutants as S81A/L82A/V84A/K92A/E96K/S129A/N172D. While the conversion of ergosterol to its glucoside was not improved, the mutant M7-1 showed the highest activity toward PPD to Rh2 with a 1,789-fold increased catalytic efficiency. Increased production of Rh2 (122-fold) was obtained through the integration of M7-1 under the control of the *P*_*TDH*3_ promoter and *T*_*CYC*1_ terminator into the *HO* locus of *S. cerevisiae* ZD-PPD-016(URA3^−^) strain in comparison with the *S. cerevisiae* ZD-PPD-016(URA3^−^) strain harboring UGT51 at 6.08 mg/L (Zhuang et al., 2017).

Similarly, the key amino acid Gln283 plays a significant role in the activity and substrate region specificity of UGTPg71A29. UGTPg71A29 is responsible for glycosylation at the hydroxyl group of C-20 of Rh1, and transfers a glucose moiety to Rd, resulting in the production of Rg1 and Rb1 in *S. cerevisiae*, respectively (Lu et al., 2018). On the other hand, the engineered *UGTPg45* could be obtained by random mutagenesis using error-prone PCR. After a screening step, the selected *UGTPg45* mutant was integrated into the X4-site of the PPD-producing strain ZW04BY-RS, resulting in the production of 60.5 mg/L of Rh2, which was 1.7-fold higher than the wild type (Wang et al., 2019). In addition, mutagenesis along with the chimeric enzyme could alter the substrate region-specificity toward PPT. UGTPg1 was shown to be region-specific to glycosylation at the hydroxyl group of C-20 of PPT to synthesize F1, which had a strong effect on three amino acid residues H144, A10, and I13 (Wei et al., 2015). In addition, amino acids H144 and H82 in the UGTPg100 enzyme catalyzed the glucose attachment into a specific position at the hydroxyl group of C-6 of PPT to produce Rh1. Interestingly, chimeric enzyme 6 (Chi_6), which possesses the N-terminal domain of UGTPg1 and the C-terminal domain, including the PSPG box of UGTPg100, could catalyze the C20-OH and C6-OH of PPT at the same time to produce F1 and Rh1. Moreover, a small amount of Rg1 was detected by incubating the Chi_6 with PPT. Subsequently, ginsenosides F1 and Rh1 were obtained through *in vivo* syntheses by introducing *UGTPg1* and *UGTPg100* into PPT-producing chassis strains.

#### Enhancing Enzyme Expression

One of the first challenges facing the development of recombinant yeasts through heterologous gene expression is the difference in biased codon usage of distinct organisms, which leads to low enzyme yields (Hu et al., 2013). Although it does not support an extreme efficiency, codon optimization plays a significant role in improving the success of enzyme expression levels by either enhancing the elongation rate of RNA polymerase, the translation efficiency, or protein folding (Parret et al., 2016; Zhou et al., 2016). For instance, it has been demonstrated that the conversion rate from DM to PPD in *S. cerevisiae* dramatically increased by 262-fold through an additional overexpression of the codon-optimized *PgPPDS* (Dai et al., 2013). Subsequently, additional codon optimization was done to enhance the biosynthesis of PPT and oleanolic acid in engineered *S. cerevisiae*: *bAS* gene from *G. glabra*, PPT synthase gene from *P. ginseng*, and oleanolic acid synthase gene from *M. truncatula* (Dai et al., 2014). Similarly, the codon-optimized genes encoding UGT74AE2 or UGTPg1 from *P. ginseng* along with DDS-fused GFP enhanced the expression levels in *S. cerevisiae* to produce two novel DM glucosides (Hu et al., 2019). Like *S. cerevisiae*, the development of recombinant *Y. lipolytica* also requires codon optimization for heterologous expression. The DDS from *P. ginseng* did not convert 2,3-oxidosqualene to DM during compound K production in *Y. lipolytica*. The integration of the synthetic codon optimization of *DDS* into single- and multi-copy sites of rDNA in the *Y. lipolytica* genome resulted in a DM production of 7.9 and 17.9 mg/L, respectively. Subsequently, the codon optimization of *PPDS, ATR1*, and *UGT1* from *P. ginseng*, was also integrated into multi-copy site rDNA of the *Y. lipolytica* genome leading to the synthesis of PPD and compound K (Li et al., 2019).

Solubility is the second hurdle facing heterologous recombinant enzyme expression in yeast. This problematic situation is caused by the partial folding and misfolding of heterologous enzymes resulting in the inclusion of body formations in the cytoplasm (Ventura and Villaverde, 2006). Many researchers solved this problem by co-expressing chaperones and transcriptional activators with related enzymes. Among various chaperones, two proteins of the endoplasmic reticulum containing the immunoglobulin heavy chain binding protein (BiP) and disulfide isomerase (PDI1) have been actively applied to improve the solubility of enzymes. While BiP belongs to the 70 kilodalton heat shock protein (HSP70) system of the chaperone to facilitate the folding and repressing aggregation of the protein, PDIs are responsible for the forming, stabilizing, and breaking of disulfide bonds in the folding process (Zapun et al., 1999; Saibil, 2013). In DM glucoside production, the titer of 3β-*O*-Glc-DM was improved by 1.43-fold after the overexpression of *PDI1* in engineered *S. cerevisiae*. In the meantime, an unfolded protein response activator, *HAC1*, was used to affect the increasing heterologous expression of enzymes in *S. cerevisiae*. As a result, the integration of genes encoding HAC1 into *3*β*-O*-Glc-DM and 20*S*-*O*-Glc-DM-producing engineered *S. cerevisiae* at the rDNA site led to 1.58- and 1.53-fold inductions, respectively (Hu et al., 2019).

#### Modulation the Expression of CYP450s

As mentioned, the expression of plant *CYP450s* is difficult in yeast because of its low solubility and misfolding in the cytoplasm. Plant CYP450s are membrane-bound enzymes that anchor in the endoplasmic reticulum of plant cells. Therefore, to increase the successful expression in yeast, plant CYP450s require a suitable redox protein for electron transfer. Another hindrance for cell growth is the overexpression of the redox partner due to oxidative stress and severe cytotoxicity (Farrugia and Balzan, 2012; Zhao et al., 2017). It has been demonstrated that the fusion between PgPPDS and NADPH-cytochrome P450 reductase (ATR1) had a strong effect on PPD production, and the N-terminal transmembrane domain had a significant effect on the efficient catalysis of the fusion system in engineered *S. cerevisiae*. Three copies of the *PgPPDS-ATR1* fusion gene were integrated into the genome of *S. cerevisiae*, resulting in a high conversion rate from DM to PPD of 98.6%. Notably, the growth of the engineered *S. cerevisiae* harboring this system was not affected by the reactive oxygen species (ROS). Finally, PPD production could be obtained at 1,436.6 mg/L in fed-batch fermentation (Zhao et al., 2016c). Using the same strategy, engineered *Y. lipolytica* harboring fusion genes *PgPPDS* and *t46AtCPR1* achieved a conversion rate of 98% of PPD from 111.8 mg/L of DM (Table 1) (Li et al., 2019).

It was demonstrated that the efficiency and activity of CYP450s were dependent on not only the ratio of each component but also the pairing efficiency between CYP450s and their redox partner. In particular, the former strategy described that CYP716Y1 from *Bupleurum falcatum* was able to catalyze the hydroxylation at C-16α of oleanane-type triterpenes. The activity of CYP716Y1 for the production of 16α-hydroxy amyrin was the highest when the ratio between CYP716Y1 and CPR1 from *A. thaliana* was 5:9 (Moses et al., 2014). The latter strategy was to boost oleanolic acid production in *S. cerevisiae* through screening the various reduction systems. Among the four reduction systems from *A. thaliana, Lotus japonicas, G. urelensis*, and *M. truncatula*, the systems with CYP716A12 and CRP from *M. truncatula* exhibited the highest coupling efficiency and boosted the production of oleanolic acid in yeast (Zhao Y. et al., 2018).

#### Synthetic Enzyme Scaffold

**Figure 4:**
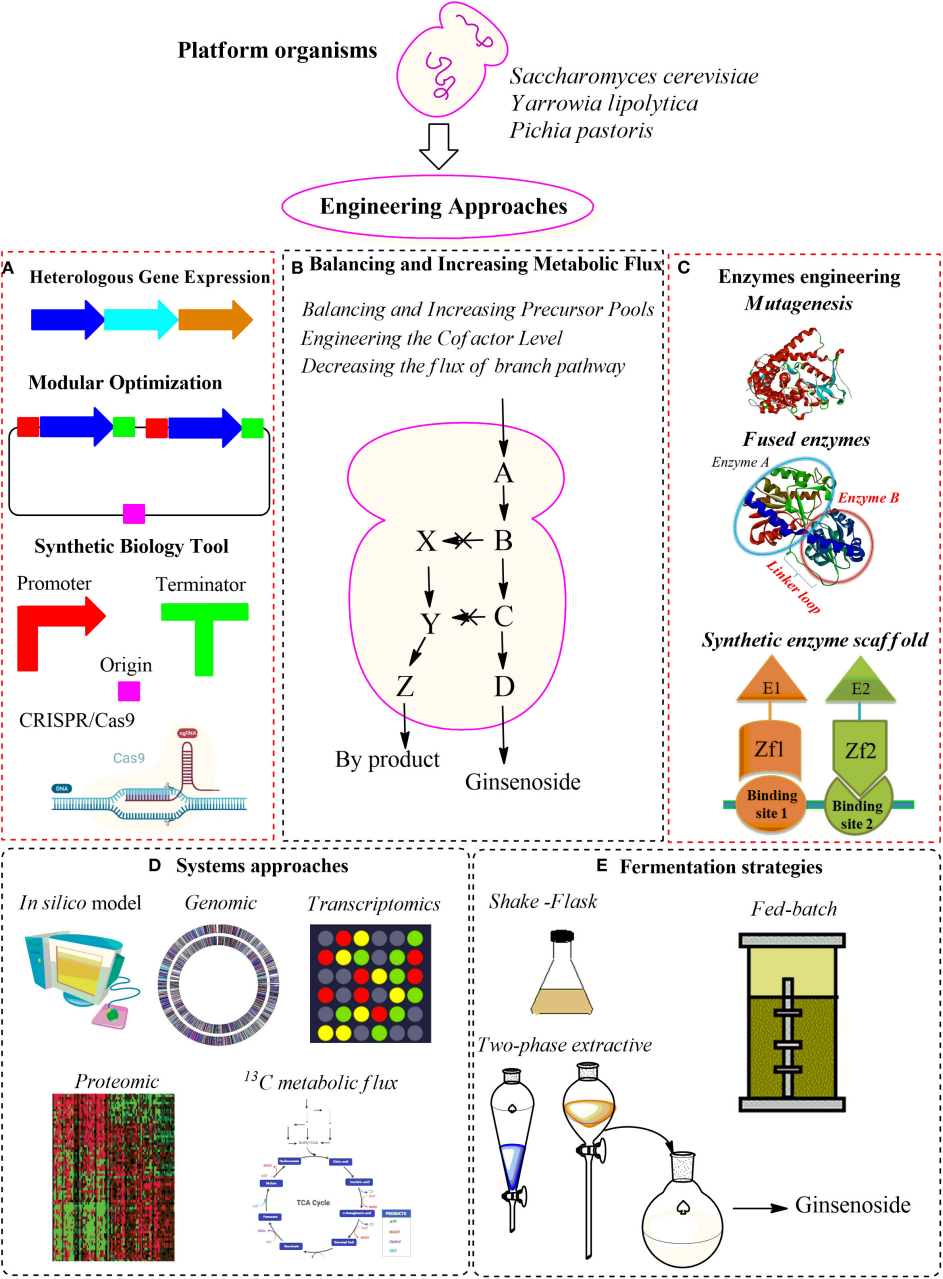
An overview of the strategies of metabolic engineering for ginsenoside production in yeasts. **(A)** Heterologous gene expression. **(B)** Balancing and increasing metabolic flux. **(C)** Enzyme engineering strategies. **(D)** System approaches. **(E)** Fermentation strategies.

Since DNA and RNA scaffolds have demonstrated a significant effect on improving the production of natural compounds, protein scaffolds have also been considered in the biosynthesis of bioactive compounds (Figure 4) (Dueber et al., 2009; Luo et al., 2015). Interactions between well-characterized enzymes and their ligands resulted in the co-localizing of spatial enzymes. Therefore, this strategy not only improves the overall pathway flux but also diminishes the metabolic burden. Enzyme scaffolds have a significant effect in increasing the effective concentration of each component in the biosynthetic pathway and in preventing the highly toxic levels of metabolite intermediates. Finally, the balancing of the relative metabolic flux using a synthetic enzyme scaffold resulted in the improvement of the target compound levels (Park et al., 2018). To produce DM in *P. pastoris*, self-assembly containing two enzyme complexes, EGR1 and PgDDS, was undertaken. While EGR1 was fused with the PDZ domain through a flexible linker L3, PgDDS was fused with a ligand of the PDZ domain (PDZlig) through a rigid 5 nm α-helical ER/K linker. The KDPEP strain for the co-expression of *PgDDS-L3-PDZlig* and *ERG1-ER/K-PDZ* with *p[PgDDS-PDZlig]/[ERG1-PDZ]* showed the highest increase with a 2.1-fold yield in DM, compared with an unassembled system (Zhao et al., 2016b).

### Systems Approaches

For heterologous gene expression in yeast systems, there are many prerequisites to identify functional genes, such as analyses of genomes, transcriptomes, and proteomes of the experimental plants. Analyses of the whole genomes of experimental plants are very useful for discovering the novel genes for the *de novo* biosynthesis pathway. Moreover, the comparison of transcriptome and metabolome data between the relative plants and platform strains observed a correlation of the cofactor and precursor pool with expression conditions for optimal ginsenoside production (Figure 4) (Seki et al., 2015; Kuwahara et al., 2019). For example, one CYP450 and four GTs have been identified as enzymes responsible for the biosynthesis of ginsenoside backbones and their derivatives in *P. quinquefolius* (Sun et al., 2010). Additionally, the transcriptome analysis of *P. japonicus* identified four genes encoding HMGR, geranylgeranyl pyrophosphate synthase (GPS), DDS, and *b*AS as key enzymes in ginsenoside biosynthesis. All the functional genes were further expressed and characterized in engineered yeast (Zhang S. et al., 2015). Recently, the MEP pathway was proved also contributing to ginsenoside production in the *P. ginseng* root (Xue et al., 2019). In particular, 2-C-methyl-D-erythritol 4-phosphate cytidylyltransferase (IspD) could be the key enzymes in the MEP pathway for ginsenoside biosynthesis. These results reveal the prospects for ginsenoside production using synthetic biology based on the MEP pathway.

^13^C-Metabolic flux analysis of *S. cerevisiae* is useful for understanding the intracellular metabolism growing in ethanol. Ethanol was used as a carbon source to supply the efficient acetyl-CoA, then carbon flux flows were mainly distributed into the glyoxylate and TCA cycle. This is because two isoenzymes, acetyl-CoA synthetase 1 and 2 (ACS1 and ACS2), were distributed in the nucleus, peroxisomes, mitochondria, and cytoplasm, and were activated to form acetyl-CoA from acetic acid when *S. cerevisiae* was grown in ethanol (Krivoruchko et al., 2015). Subsequently, the high levels of acetyl-CoA resulted in the improvement of PPD biosynthesis (Zhao F. L. et al., 2018). In addition, ^13^C-metabolic flux analysis of *S. cerevisiae* revealed that PPD biosynthesis from ethanol required 18 mol ethanol and 3 mol NADP^+^ along with 54 mol ATP as follows: 18 ethanol + 3 NADP^+^ + 18 NAD^+^ + 54 ATP + 6 O_2_ + 6 H_2_O → 1 PPD + 18 CoA + 3 NADPH + 18 NADH + 54 (ADP + Pi + 6 CO_2_) (Zhao F. L. et al., 2018).

### Fermentation Strategies

After the successful construction of ginsenoside-producing chassis strains, creating the optimal conditions for large scale operations is important to improve production levels. Several factors have significant effects on productivity, including media, pH, temperature, and carbon source (Table 1). In general, a yeast extract-peptone-dextrose (YPD)-rich medium was used as the common media for ginsenoside production in yeasts. For example, 21.4 mg/L of oleanolic acid was obtained from the engineered *S. cerevisiae* GY-1 under growth in YPD medium (Dai et al., 2014). Similarly, *Y. lipolytica* YL-MVA-CK grown in YPD medium produced 161.8 mg/L of compound K, while DM achieved 0.10 mg/g DCW by *P. pastoris* KDPEP (Zhao et al., 2016b; Li et al., 2019). Additionally, the synthetic complete (SC) media was also applied for ginsenoside production. Rh1 and Rh2 were produced in SC medium by *S. cerevisiae* ZW-Rh1-20 and M7-1 with 98.2 and 300 mg/L, respectively (Wei et al., 2015; Zhuang et al., 2017). Furthermore, using synthetic defined (SD) media obtained 1,189 mg/L of PPD in ZD-PPD-018 (Table 1) (Dai et al., 2013).

The optimal pH and temperature have been reported to be 5.5 and 30°C for ginsenoside production in the engineered *S. cerevisiae* (Dai et al., 2014; Zhao F. L. et al., 2018; Hu et al., 2019). Two yeast strains, *Y. lipolytica* and *P. pastoris* had an optimal pH of around 6.0 (Zhao et al., 2016b; Wu et al., 2019). Glucose has previously been used as the main carbon source for ginsenoside production in yeast. Recently, ginsenoside biosynthesis in yeast has been generated from alternative carbon sources, such as ethanol, methanol, galactose, and xylose.

Although methanol was used to induce gene expression under the control of the methanol-induced *P*_*AOX*1_ promoter, methanol and glucose might be used as dual carbon sources for DM production in *P. pastoris* KDPEP (Zhao et al., 2016b). Shake flask cultivation of *S. cerevisiae* IN-B produced 9.05 mg/L of 3β,12β-Di-*O*-Glc-PPD from galactose (Liang et al., 2017). In addition, the engineered *Y. lipolytica* 14 was able to produce PPD from xylose with 300.63 mg/L, which was 1.8-fold higher than using glucose as a carbon source (Wu et al., 2019). Interestingly, co-fermentation of glucose and xylose by *S. cerevisiae* GW6 obtained a higher PPD titer and yield in comparison with only using glucose as a sole carbon source (Gao et al., 2018).

Since reactive oxygen species (ROS), which were released by PPDS-CPR uncoupling and ethanol stress, causing negative effects on cell viability as well as metabolism, enhancing the tolerance ability of yeast was considered for ginsenoside production in the fermentation process (Ohta et al., 2016; Zhao et al., 2017). Several strategies have been reported to overcome these disadvantages for improving PPD production in *S. cerevisiae*. First, the construction of an integrated cell wall was conducted to improve resistance to high-level ethanol, which could prevent cytotoxicity from disrupting cell growth (Ohta et al., 2016). Second, the consumption of intracellular ROS was carried out by the overexpression of the genes encoding oxidative stress response regulator (Gulshan et al., 2011). In particular, the robust *S. cerevisiae* W3a-ssPy harbored suppressor of SIT4 deletion 1 (*SSD1*) gene encoding proteins involved in improving cell wall integration and Yap1-binding protein (*YBP1*) gene encoding proteins involved in degrading intracellular ROS, which decreased the ROS level to 75.2% and improved cell growth from 73.6 to 86.3%. Subsequently, the engineered strain was used to produce PPD with 4,250 mg/L based on glucose-ethanol carbon source stage-controlled fermentation (Zhao et al., 2017).

A solvent-free system containing n-butanol and methanol-acetone was used for the ginsenoside extraction from engineered yeast. However, the accumulation of a black solid in the fed-batch fermentation was not removed (Wang et al., 2019; Wu et al., 2019). Therefore, two-phase extractive fermentation was used to extract ginsenoside *in situ*, resulting in the improvement of the final product. It was demonstrated that two-phase solvents, including dodecane and methyl oleate, had a significant effect on the production of PPD and DM. While dodecane was added to the fermenter to immediately remove foams, methyl oleate was able to inhibit the formation of black solids. Finally, the total PPD and DM in both the cells and solvent were achieved at 1,189 and 1,548 mg/L, respectively (Dai et al., 2013).

## Conclusion And Future Prospective

Ginsenosides exhibit wide benefits in nutrient additive and pharmacological properties. To achieve the efficient production of ginsenosides in yeast, various metabolic engineering techniques have been developed in recent years, such as heterologous gene expression, balancing, and increasing metabolic flux, enzyme engineering, and systems approach along with fermentation strategies (Figure 4). Although the titer of ginsenosides could be increased by balancing and increasing the supply of the precursor acetyl-CoA and cofactor NADPH, the modulation of the biosynthesis pathway genes expression and protein engineering are preferred the application in yeast. Combining all engineered strategies is necessary to optimize the yeast cell factory for ginsenoside production. The titer of PPD could achieved up to ~11,017.2 mg/L by overexpressing all MVA pathway genes and optimizing the expression level of cytochrome P450 enzymes. This is the highest titer of PPD ever obtained in a lab-scale fermentation. Based on this PPD-producing chassis strain, the optimization of expression level and activity of UGTPg45 resulted in the highest ginsenoside Rh2 titer up to the present with 2,252.3 mg/L in 10 L fed-batch fermentation (Wang et al., 2019). While UGTPg45 expression level increased by increasing its copy numbers and engineering its promoter, activity of UGTPg45 in engineered yeast improved by direct evolution and screening for novel UGTs with higher C3-OH glycosylation efficiencies from other plant species.

Combining all engineered strategies could be obtained up to more than 11 g/L, however, the potential ginsenoside biosynthesis in yeast remains large capacity. Firstly, the other suitable yeasts should be expanded as promising hosts. The oleaginous yeast, *Y. lipolytica* belongs to the high flux through acyl-CoA precursors and the TCA cycle for heterologous terpenoid synthesis (Abdel-Mawgoud et al., 2018). The methylotrophic yeast *P. pastoris* not only showed the ability to use methanol as a carbon and energy source but also presented high NADPH generation per carbon (Pena et al., 2018). Recently, the available full genome sequences of *Y. lipolytica* and *P. pastoris* resulted in increasing the number of amplifications of these strains for ginsenoside production. Unlike *S. cerevisiae*, the growth ability of wide pH ranges of both strains makes them more favorable hosts for ginsenoside production (Pena et al., 2018; Wu et al., 2019). Secondly, to construct a novel platform for ginsenoside production, the exploitation of novel key genes, such as *CYP450s* and *GTs*, is required. The efficient high throughput method and a biosensor sensing ginsenosides are also required to develop for novel enzyme exploitation. The strategy allows engineered yeasts to generate unnatural ginsenosides. Subsequently, the improvement of the efficient enzymes *via* directed evolution and rational design enzyme also represent great promises. Thirdly, metabolic engineering requires strong links with genomic-, transcriptomic-, proteomic-, phosphoproteomic-, and bioinformatic-guided synthetic system approaches for the deep learning of ginsenoside biosynthesis in different yeasts. This will facilitate the optimal modular with multiple gene expression, resulting in ginsenoside productivity in host strains. Fourthly, the development of the RNA interference (RNAi) or CRISPR-Cas9 systems for engineering yeasts opens promising host strains with high titers of ginsenosides. CRISPR-Cas9 tool can perform quickly and facilitates knock-down, knock-out, and knock-in of large DNA fragment, which is important for developing of the efficient cell factories. Fifthly, it should be noted that the transporters have a significant effect on transferring ginsenosides from cytosol to the extracellular location. Therefore, crystal structures and understanding the mechanisms of transporters are required to reduce the cytotoxicity of ginsenosides to yeast strains. Finally, the development of yeast capable of fermenting mixed sugars simultaneously from renewable biomass is necessary for the industrial-scale production of ginsenosides. Again, yeasts are promising chassis strains for ginsenoside biosynthesis.

## Author Contributions

LC conceived the idea, surveyed the literature, prepared figures, drafted manuscript, and revised the manuscript. JM and HB revised the manuscript. HB gave valuable suggestions. All authors read and approved the final manuscript.

### Conflict of Interest

The authors declare that the research was conducted in the absence of any commercial or financial relationships that could be construed as a potential conflict of interest.
